# Black women diversity leaders' perceptions of organizational inclusivity in college sports

**DOI:** 10.3389/fspor.2022.923649

**Published:** 2022-11-01

**Authors:** Ajhanai Channel Inez Keaton

**Affiliations:** Department of Health and Sport Sciences, University of Louisville, Louisville, KY, United States

**Keywords:** intersectionality, Black women, college sport, diversity leaders, intersectional invisibility, inclusivity

## Abstract

Select collegiate athletic departments have adopted Athletic Diversity and Inclusion Officer (ADIO) positions. ADIOs are formally tasked with the job responsibilities of creating diverse, equitable, and inclusive athletic departments, and many individuals holding the positions are Black men and women. This hermeneutic phenomenological study focused on the leadership of Black women in ADIO positions and examined how their racial and gender identity informed their perceptions of organizational inclusivity. Findings reveal that the intersecting identities of Black women are drawn upon and centered to make sense of what organizational inclusivity is. More specifically, organizational inclusivity is creating contexts that do not mirror Black women's experiences as *outsiders within* mostly White athletic departments, lived experiences entangled in systems of oppression, specifically sexism and racism (read: intersectionality), and experiences that cultivate Black feminist thought in Black women, as this consciousness is only developed through adverse realities of exclusion. Hence, Black women ADIOs' perception of organizational inclusivity is informed by their own intersectional lived experiences of exclusion in sports and society writ large.

## Introduction

Recent media depictions of Black women participating in sports (1) and representational data of Black women leading sports organizations (2) illuminate how Black womanhood is entangled in the patriarchal and racist systems (3). Sports, like many other United States institutions (e.g., housing, education, etc.), is upheld by inequitable structures and ideologies (4, 5), which heightens the raced-gendered marginalizing realities of Black women and unfortunately provides sports fans a lens for making sense of the inequities Black women experience in sports (1, 6). Consequently, sports offers a lens for studying Black women's relationship with sexist and racist structures (7). For example, Candance Story, Athletic Director of Vanderbilt University, is celebrated for being the first Black and first woman Athletic Director in the school's history and the second Black woman in the history of the commodification of big-time collegiate athletics to lead a major (read: wealthy/athletic prestige) athletic department (8). However, such an accomplishment is a striking example of Black women navigating systems of patriarchy and racism in National Collegiate Athletic Association (NCAA) hiring practices (4, 9). Similarly, Dawn Staley, Head Coach of the University of South Carolina Women's Basketball team, earned an impressive contract extension in 2021, which states she is set to earn 22 million dollars over a seven-year period (10). Coach Staley is rightfully being paid her worth (given all she has done to uplift women's basketball), but we cannot ignore how Black women are systemically barred from attaining the head coach position in women's basketball at the Division I level (11, 12), given that Black women account for only 18% of head coach positions, while their White female counterparts account for 45% (2). Therefore, the historic and groundbreaking careers of Candance Story and Dawn Staley are not indicative of Black women in college sports due to the institutional field being a racialized (4, 9) and deeply gendered organization (13–15).

Given that Black women do not hold many positions of power in NCAA college sports administration across the three divisional levels (2), their status in an emergent collegiate sports leadership position is noteworthy. Black women are currently holding many of the novel Athletic Diversity and Inclusion Officer (ADIO) positions in Division I athletics (4, 16). An ADIO, not to be mistaken for an Athletic Diversity and Inclusion Designee [see (17)], is an administrative position *formally* tasked with the job responsibilities of leading an athletic department's diversity, equity, and inclusion (DEI) aims (16). The adoption of ADIOs is an extension of the growing urgency extant in college sports to ensure DEI is incorporated into their organizational contexts (4, 16, 18). While these intentions are commendable, Keaton (16) argues how these aims are borne out of a desire for *legitimacy* [see (19)], as athletic departments continue to uphold ideologies of abstract liberalism and refrain from addressing how deeply embedded racial injustice is to their structures and practices (4, 20).

Moreover, given the adverse realities of some Black women college sports leaders (11, 12, 21), this scholarship is concerned with how Black women lead the charge of creating more inclusive organizations, given the institutional field (e.g., NCAA collegiate athletics) they navigate and lead within is complicit in upholding sexist and racist practices and structures (4, 9, 13). Black feminist scholars and Black feminist epistemologies perceive Black women to be experts of their social world (22, 23). Collins (24) asserts that “Black women have a self-defined standpoint on their own oppression” (p. 747). Drawing upon this epistemological stance, I sought to understand how the raced-gendered identity of Black women ADIOs informs their perceptions of organizational inclusivity as diversity leaders of sports organizations. This study provides insights into how Black women ADIOs' perceptions of organizational inclusivity are informed by their intersectional lived experiences. It advances the field of Sports Management and Sports Sociology, as it is the first study grappling with how Black women's “standpoint on their own oppression” is drawn upon to conceptualize organizational inclusivity as a diversity and inclusion officer in sports organizations. Consequently, their intersectional lived experiences (25, 26) as Black women become centered to make sense of what organizational inclusivity is, which are organizational contexts inclusive to Black women and contexts that do not mirror Black women's intersectional marginalized experiences in sports and society writ large. More specifically, organizational inclusivity is creating contexts that do not mirror Black women's experiences as *outsiders within* mostly White athletic departments, lived experiences entangled in systems of oppression, specifically sexism and racism (read: intersectionality), and experiences that cultivate Black feminist thought in Black women, as this consciousness is bolstered through adverse realities of exclusion. The following sections provide an overview of the literature on Black women sports administrators holding college sports leadership positions, discuss prominent Black feminist epistemologies, introduce the theoretical lens guiding this work (intersectionality), and offer critical attributes of the methodology (interpretative phenomenology) and the data analysis technique (interpretative phenomenological analysis) deployed.

## Background

### Black women and intercollegiate sports leadership positions

The NCAA Division I collegiate athletics is a racialized organization (4) and a deeply gendered organizational field (13, 14). The leadership experiences of Black women sports administrators are reflective of navigating racist and sexist structures and practices (11, 21, 27). These Black women college sports administrators (21, 27) and coaches [Borland and Bruening (12)] are cognizant of how their adverse raced-gendered treatment has less to do with their competencies and abilities in their leadership positions and more to do with their non-prototypical identities as White and male identities are prototypical identities in prominent collegiate sports leadership positions (2).

Black women assistant basketball coaches perceive their racial and gender identity as hindering their career ascension (11). Similarly, collegiate sports administrators in Price et al. (27) perceive their raced-gendered identities as leading to difficulties being understood by White male athletic directors (ADs), who usually influence and control the hiring process. Although Black women ADs in historically Black colleges and universities (HBCUs) hold a dominant racial identity (i.e., Black) in these organizational contexts, their gender identity makes these women targets of not only sexism and gender stereotypes but also raced-gendered stereotypes specific to Black women, like *The Angry Black woman* (21). Hence, Black women leaders in varying collegiate sports leadership positions (e.g., athletic director, coach, etc.) and institutional types describe their experiences as being heavily influenced by their social identities, and they are cognizant of how being a Black woman in sport organizations has led to marginalizing experiences in hiring practices, career ascension, navigating adverse organizational cultures, and experiencing pressure to conform or limit self (4, 12, 21).

### Theoretical framework: Black women intersectional lived experiences

A prominent Black feminist epistemology is that Black women have a distinctive consciousness, often referred to as Black feminist thought (BFT), which enables them to interpret and make sense of their social environment in a manner that is disparate from those of other varying identity groups (11, 24). Black feminist thought is concerned with how Black women develop alternative ideas of self, Black women collectively, and the inequitable structures of their social world (24). Black women who draw upon BFT can navigate not being constrained by their “both/and” status and come to use the knowledge of their own oppression to make sense of their *outsider within* status [(28), p. 771]. There are three key themes of BFT: (a) Black women's thought is in concert with historical and material conditions that inform their perceptions of their social world, (b) Black women's unique perceptions of their social world share a similar interpretation with other Black women, and (c) Black women's disparate identities on the axis of class, sexuality, region, and age inform how BFT is expressed (28).

Black feminist thought finds a connection to Black women's *outsider within* status. This Black feminist concept is commonly associated with Collins (29), a seminal piece of Black feminist scholarship. Ransby (30) asserts that an “*outsider within* has the benefit of observation up close, but she is still not an authentic member of the inner circle…” (p. 370). However, precursory to Collins (29), Black women have long been attuned to their positionality as an *outsider in* White spaces (31, 32). For example, consider the Black women domestic workers who worked and cared for White families and were conceptualized as “family” by many White people in the mid-20th century (31). Despite being perceived as “family,” these Black women domestic workers knew they would never *truly* be accepted by the White families they worked for, so they remained *outsiders*. Thus, Black women can be *in* White spaces (e.g., working in White households, White dominated organizations), but they still operate on the margins of power and experience marginalization. The *outsider within* status of Black women strengthens their BFT because they learn/study the epistemologies of spaces that have excluded them, while still holding onto how their consciousness is historically and contemporaneously in concert with their lived experiences (29).

### Intersectionality

Crenshaw (25) is the pioneering work that provided succinct language for Black women being simultaneously marginalized at the intersection of race, gender, and other social identities like class (33, 34). However, Black women scholars, activists, mothers, caregivers, etc., have always been attuned to these social dynamics that encompass what we contemporarily understand as *intersectionality* (23, 32, 35). Consequently, credit is due to Crenshaw (25) for giving academics the terminology to succinctly make sense of Black women's lived experiences operating in tandem in multiple systems of oppression. Nonetheless, intersectionality is rooted in Black feminist epistemologies, and the terminology is borne out of critical legal studies. Black women were unable to successfully win legal suits on grounds of gender or racial discrimination (25). Hence, Black women failed to be perceived as fully *woman* and fully *Black*. This race-gendered tension illuminated how in the case of Black women, gender and racial discrimination co-exist to embody their lived experiences and concomitant oppression.

Scholarships applying intersectionality in sports management have brought attention to how the sexism and racism rife in Division I collegiate athletics has halted the leadership ascension of Black women assistant coaches and ADs simply because they are Black women or Black lesbian women [Borland and Bruening (12)]. This scholarship demonstrates how systems of patriarchy, racism, and homophobia force Black women to conceal their sexual orientation in a manner that differs from White lesbians (12), and Black women ADs are unjustly navigating their athletic departments in a manner that does not align with the power of their position (21). Consequently, previous scholarship has used intersectionality as a form of *critical inquiry*, but as a scholarly community, we have yet to see intersectionality being used as both *critical inquiry* and *critical praxis* (26) in sports management.

Collins and Bilge (26) assert that “intersectionality is not simply a method for doing research (*critical inquiry*), but also a tool for empowering people (*critical praxis*)” (p. 43). They argue that the full utility of intersectionality has yet to be achieved by many academics, as our application of the framework is often applied only as a *critical inquiry* tool. Using intersectionality as a tool of *critical inquiry* means applying intersectionality as a theoretical framework. Hence, intersectionality guides the study, interview questions, and analysis. However, using intersectionality as a tool of *critical praxis* means that intersectionality is used to not only better *understand* social problems (*critical inquiry*) but also solve social problems (*critical praxis*). As it relates to the sports management discipline, I agree with the proclamation of Collins and Bilge (26) that sports scholars can be more intentional in applying the epistemological underpinnings of intersectionality in a manner that captures *critical praxis* displayed in scholars and research participants. According to Collins and Bilge (26), “problem-solving lies at the heart” of using intersectionality as *critical praxis* (p. 50). This work seeks to engage in *critical inquiry* and *critical praxis* to ensure we as a discipline use the full utility of intersectionality.

### Intersectional invisibility

Aligning with the arguments of Crenshaw (25), the work of scholars in the field of management and social psychology captures how Black women navigate their social world entangled in systems of marginalization (36–38). However, they question if there are instances, contexts, and opportunities whereby Black women can experience distinctive advantages due to holding non-prototypical identities (36). In short, the aforementioned scholars bring attention to how Black women being non-prototypical in White and male-dominated spaces (like sports organizations) leads to experiences of invisibility. Purdie and Eibach (36) have coined these instances as *intersectional invisibility*. Intersectional invisibility states individuals who do not hold dominant American societal identities (White, man, heterosexual, etc.) are non-prototypical in particular contexts. Black women are non-prototypical in White and male-dominated spaces (e.g., collegiate athletic administration), and this status can position them as invisible or unfamiliar (37).

Smith et al. (38) found that Black executive women (e.g., University Deans, Chief Executive Officers, Fortune 500 Vice Presidents, etc.) perceived their intersectional invisibility status to weaken or “cancel” out the impact of gender and race marginalization in their professional experiences – referred to as *benign intersectional invisibility*. Benign intersectional invisibility challenges prescriptions of Black women conditionally being in a state of marginalization. Participants in Smith et al. (38) perceived being non-prototypical in their organizations to bolster how they made connections with clients of diverse backgrounds, encouraged them to bring their authentic selves to the workplace, and created opportunities for them to display cultural competence (38). Although participants experienced benign intersectional invisibility, adverse organizational experiences of race-gendered marginalization or *hostile intersectional invisibility* (e.g., stereotypes, exclusion, silencing), was prevalent in their experiences as well (38). These participants discussed their marginalization and how they were perceived as unqualified in the executive positions they attained because of their non-prototypical identities in their workplaces.

Consequently, the theoretical prescriptions of intersectional invisibility (36, 38) and intersectionality (25) inform this scholarship because we are unaware of how the ADIO position may enable these Black women ADIOs to have disparate organizational experiences in comparison to Black women coaches (11) and athletic directors (21). Given there has yet to be an empirical study on how the intersecting identities of Black women ADIOs inform their perceptions of organizational inclusivity in sports organizations, this study acknowledges that Black women administrators report adverse organizational experiences in college athletics (12, 27), while also considering how the novelty of the position (16) possibly creates sentiments of benign and hostile intersectional invisibility (38).

## Research methodology

### Interpretative phenomenology

This hermeneutic or interpretative phenomenological study examined how the intersecting identities, specifically race and gender, inform Black women ADIOs' perceptions of organizational inclusivity. Phenomenological research seeks to make sense of lived experiences by studying phenomena intimately with attention being given to the *meaning* of lived experiences (39–41). Hence, phenomenologies are less concerned about solving social problems and study the essence of lived experience with the intent of becoming deeply familiar with an individual's reality (42, 43). Interpretative phenomenology emphasizes that scholars interpret human experience rather than simply describing participants' experiences (44, 45). This means that an interpretative phenomenological study should not silence the voice of scholars or their lived experiences, thus *bracketing* is not necessary, but it would be necessary for a descriptive or Husserl phenomenological examination (45, 46).

There are key attributes of interpretative phenomenological research: (a) The interpretation of lived experiences has semantic and layered understandings, (b) our existence as humans is in concert with the peculiarities of society, and (c) interpretation is a foundational aspect of the human experience (39). Scholars engaging in interpretative phenomenological research are making sense of how participants make sense of their lived experiences (44). Such an essential premise finds a connection to Heidegger's concept of *co-constitutionality*, which asserts “…that the meanings the researcher arrives at in interpretive research are a blend of the meanings articulated by both participant and researcher within the focus of the study” [(47) p. 730]. This co-creation of meaning-making enables researchers to bring self into their work and perceives participants as experts of their lived experiences. However, it is important to note that *co-constitutionality* does not require that a scholar's meaning-making is *in congruence with participants*, but the meaning made of the experience should be evident in the lived experiences shared by participants (47).

### Sample

As of August 2020, NCAA institutions are required to designate the role of Athletic Diversity and Inclusion Designee, commonly referred to as ADID, to an administrator affiliated with NCAA athletic departments (16). ADIDs hold a primary position and are assigned varying ADID-related tasks. Hence, the advancement of DEI can be understood as an “extra” responsibility, given that an ADID is not a position but a designation. This research studied Athletic Diversity and Inclusion Officers commonly referred to as ADIOs (16). An ADIO is an administrative position that is formally and primarily responsible for advancing DEI in sports organizations, and this position more closely resembles Chief Diversity Officers in corporate and business domains (16).

The population of ADIOs is sparse but growing as an influx of position adoptions occurred following the murder of George Floyd and during an increased tenure of athlete activism (4). Given the timeliness of this study and the research methodology, five Black women participated in this study. Phenomenological research encourages smaller samples and purposive sampling techniques (44, 47). Hence, participants were purposively contacted *via* email and met the following criteria: (a) identified as a Black woman, (b) held the ADIO position for at least 3 months, (c) their position title included some semblance of language around diversity, inclusion, and equity, and (d) they currently work for a Division I institution. Given the ADIO position is in an emergent state (16), my intent was not to interview as many Black women ADIOs as possible, as doing so could have created issues of anonymity and possibly hindered my ability to create trust amongst participants, as some did have concerns regarding how their identity would be protected. Lastly, I engaged in this research as a critical and Black feminist scholar, and if increasing my sample would jeopardize or adversely contribute to the experiences of my participants, then *more participants* does not align with my purpose or scholarly identity.

To protect participant confidentiality, the exact position title, geographical location, educational background, and tenure in the ADIO position are not presented. The participants are as follows: Serenity, Monique, Nia, Kayla, and Jalyiah. These names are pseudonyms.

### Data collection

Serenity, Monique, Nia, Kayla, and Jalyiah participated in two semi-structured in-depth interviews and completed two reflective journal prompts. The second interview was scheduled at least a week after the initial interview. After the first interview, participants stated that they have never been asked these “types of questions before” or “never really thought about how their identity was relevant to the ADIO position.” Once each interview was completed, participants continued to discuss the complexity and nuances of being tasked to create more inclusive athletic departments, even though they continue to experience marginalization themselves. I share these insights to illuminate how valuable each interview was not only for the purpose of this study but also for the participants, as these interviews were described as being “cathartic.”

Individual interviews were collected during the COVID-19 pandemic, recorded *via* Zoom, a video conference platform, and lasted between 60 and 90 min. I utilized the reflective journal prompts as a tool to allow participants more time to reflect upon their lived experiences, as a tool to ask participants follow-up questions, and as an opportunity for participants to clarify and expand upon their interview commentary.

### Data analysis

I conducted an interpretative phenomenological analysis (IPA) (44). IPA is an analysis method that centers interpretation, hermeneutics, and idiography (48) and aligns with the philosophical prescriptions of Heidegger's interpretative phenomenology (44). IPA analyses seek to illuminate the *meaning* of lived experiences and center on how the meaning made is co-created with participants (39, 44). The interpretation attribute of IPA moves beyond stating what a participant experiences and calls for researchers to interpret what these lived experiences *mean* (44). The hermeneutics attribute of IPA is the practice of *double hermeneutics*, the convoluted process of researchers making sense of what participants themselves are attempting to make sense of (48). Idiography addresses how researchers must be attuned to the particular of their research participants (44, 48). This investigation is rooted in such a principle that I was concerned about the peculiar reality of Black women creating inclusive sports organizations, known for racial and gender marginalization (4). In terms of the analysis, idiography is relevant to how researchers question the data and the circumstances of participants' lived experiences. Lastly, there is not a prescribed structure for IPA, but interpretation, hermeneutics, and idiography must be central to the process (44).

After each individual interview, I immediately engaged in memoing, specifically on the following: (a) my initial thoughts, (b) rememberable comments, (c) statements that challenge preconceived notions I held, (d) blatant commentary about being a Black woman ADIO, and e) my reflections on how being in this space with participants made me feel. Engaging in this process led to me organically participating in interpretation and idiography. While engaging in this immediate post-interview memoing, I would ask myself “why questions,” like (a) Why was Monique able to laugh while sharing hostile stories of raced-gendered exclusion, (b) Why does Nia continue to stay in her organization if she feels silenced, and (c) Why is Serenity comparing her leadership to Stacy Abrams? As I posed these questions to myself, I was grappling with the meaning of these lived experiences while also being attuned to “the particular,” like laughter, the language use of “silenced,” and the peculiarity of an ADIO comparing themselves to a contemporary Black woman political figure (e.g., Stacy Abrams). Consequently, my immediate (and continued) memoing centered on meaning-making, interpretation, hermeneutics, and idiography, which are critical aspects of interpretative phenomenology and IPA.

I then transitioned to coding each participant's lived experience individually before examining how participants held congruent and divergent experiences (44). Consequently, I established participants' unique individual themes before transitioning to establishing themes for another participant. Doing so allowed me to focus on one person while suspending the lived experiences of other participants. However, my first round of initial coding did not begin until I read and listened to interview transcripts at least two times, as I sought to enter the coding process being very familiar with the data. I began the initial coding process by applying concept codes (e.g., “intersectionality,” “whiteness,” “bricolage”) (49), descriptive comments (44), and linguistic comments (e.g., attentive to stutters, pauses, laughing, and deep breathes) (44). After establishing these initial codes, I engaged in abstraction, the process of organizing similar codes to create second-level codes (44). Second-level codes consisted of “Knows Marginalization,” “Intersectional Benefits,” and “Identity Entanglement.” Once these second-level codes were established, I examined if the excerpts attached to these codes held a similar interpretation and *meaning*. Consequently, a shared code did not equate to a shared interpretation, which led to me engaging in the iterative process of creating new codes/engaging in more analysis, which eventually led to the theme development centered on *meaning*. After completing this process for each participant, I examined how participant themes diverged and converged and examined if shared themes held a similar interpretation, which led to the creation of three superordinate themes.

## Findings: Intersecting identities and perceptions of organizational inclusivity

As it relates to the research question (How do the intersecting identities of Black women ADIOs inform their perceptions of organizational inclusivity in their respective sports organizations?), Black women ADIOs draw upon their experiences of organizational exclusivity [Borland and Bruening (12, 50)] and societal marginalization (23, 24, 35) to inform their perceptions of organizational inclusivity. Because Black women experience the simultaneity of sexism and racism (25), prominent marginalizing systems in collegiate athletics (4, 9), participants state being cognizant of what exclusion *looks* and *feels* like. Black women ADIOs use these experiences of raced-gendered marginalization to inform their perceptions of organizational inclusivity, illuminating how they center their “own oppression” [(24) p. 747] to inform their perceptions of organizational inclusivity as an ADIO.

**Figure 1:**
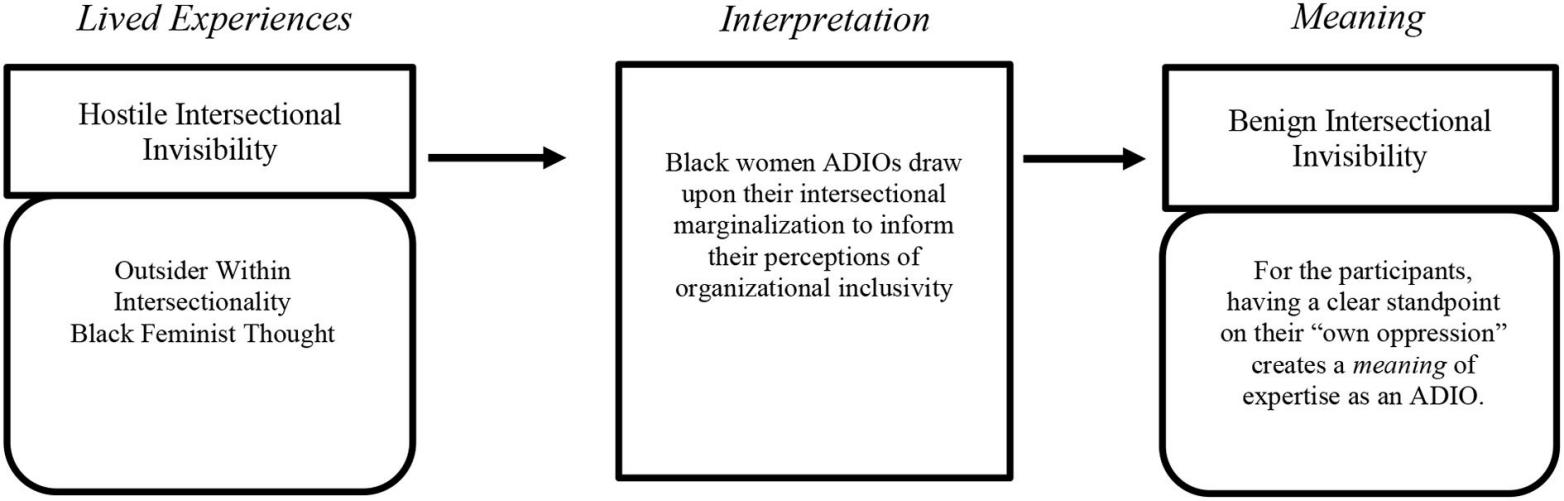
Meaning making of organizational inclusivity in black women ADIOs.

As evident in Figure 1, Black women ADIOs specifically discuss how their experiences as an *outsider within* mostly White athletic departments, their lived experiences in marginalizing systems, and their distinctive lens as a Black woman (Black feminist thought) inform their perceptions of organizational inclusivity. Consequently, the aforementioned realities are marginalizing experiences due to Black women navigating systems of patriarchy and racism (25, 28) and experiences that capture *hostile intersectional invisibility* (38). Moreover, participants having a clear standpoint on their “own oppression” [see Collins (24) p. 747] creates a *meaning* of expertise or matters of *benign intersectional invisibility* (38) to inform their perception of organizational inclusivity as Black women ADIOs. For the participants in this study, organizational inclusivity is creating contexts that are inclusive to Black women.

### Outsider within: Experiences on the margins of power to make sense of organizational inclusivity

Participants discussed how their professional experiences in collegiate athletics have relegated them to navigate being on the margins of power (30) due to their raced-gendered identities being non-prototypical identities of collegiate sports leadership (2). This experience as an *outsider in* predominately White athletic departments makes these leaders familiar with what exclusion *feels* and *looks* like. In turn, they sought to create inclusive organizations that do not mirror their *outsider within* experiences (29, 30), which is the concomitant reality of being “the only one,” “silenced,” and “disrespected.” Being “the only one” or one of few Black women among mostly White administrators is an isolating experience and Black women ADIOs recall what it *feels* like to be an *outsider within* and center this feeling to conceptualize organizational inclusivity. Monique shares:

I have those [only one] experiences to reflect on and when I'm in meetings with other people (non-Black women), you know, there's times they (non-Black women), haven't been the only one in the room that looks like them. And what has happened to me in the past, allows me to, no it forces me to think about everyone else in a way that I wish people would have thought about me.

Monique's *outsider within* lived experience “forces” her to think of others. Her use of “forces” emphasizes how she cannot refrain from incorporating her experiences as a Black woman into her perceptions of organizational inclusivity. Additionally, she perceives Black women as having a distinctive insight, because “they,” meaning individuals who are not Black women, rarely experience such an isolating experience. Although being the only Black woman in collegiate sports spaces creates an *outsider within* reality (29), Black women ADIOs carry these lived experiences into their leadership to make sense of inclusivity for others. The isolation attached to being an *outsider within* has created sentiments of feeling “undervalued” and “disrespected,” as they navigate being on the margins of power. Jalyiah leans into these contemporary and previous adverse feelings to center on how she considers the marginalization of others. Jalyiah shares:

I know the feeling of being undervalued, where it's really not being heard, where it's feeling disrespected, overlooked. Um, just, you know, the- the-the pick the, pick the descriptions like we've had that experience. We've (Black women) had the inequity, the gaps, the discrimination, the oppression, like we felt all those things. So that- that experience as a Black woman can almost translate into the- the experiences of all these other underrepresented groups.

Jalyiah begins by stating all the adverse concomitant feelings and experiences of being an outsider within – “disrespected,” “overlooked,” “discriminat[ed],” and “oppress[ed].” However, she concludes her thought by stating how these experiences are relevant to other “underrepresented groups.” Hence, her positionality as an *outsider within* (29, 30), while not an ideal experience, is an experience that enables Jalyiah to consider how Black women are not the only individuals with lived experiences on the margins of power. For Jalyiah, the concomitant feelings of being an *outsider within* enable her to perceive organizational inclusivity as pertinent to “underrepresented groups” not solely her or Black women. Thus, Black womanhood, specifically the *outsider within* experience, is used as a barometer for making sense of exclusivity and a tool drawn upon to create empathy in Black woman ADIO leadership as their perceptions of organization inclusivity center on the marginality of other “underrepresented groups” in collegiate athletics, because she is familiar with what it means to exist on the margins. Jalyiah continues to elaborate:

Um, and I know what it's like to just not belong. And not feel like you're heard and not feel like you're valued and just like not fit in. And what makes me respond [read: seek inclusivity] is when, it, it, it's like almost like a trigger, it's like, I know that the way someone says something [hurtful] will lead to, or if someone's acting in a [hurtful] manner will proceed to someone feeling that way (e.g., “not belong”).

She continues to use her adverse lived experiences (read: hostile intersectional invisibility) on the margins of power and the concomitant feelings of her own exclusion (e.g., “heard but not valued”) to influence how she “responds” as an ADIO. As an ADIO, her “respond[ing]” to exclusivity or marginalizing conditions or incidents is reflective of her perception of organizational inclusivity, as her need (“trigger”) to respond signals that addressing organizational inclusivity is an intentional effort. Additionally, Jalyiah equating her “respond[ing]” to exclusivity as a “trigger” demonstrates how her perception of organizational inclusivity is based upon her own lived experiences. She reacts like a “trigger” (read: swiftly, promptly) because she is familiar with how individuals in positions of power attempt to keep her on the margins or outside. This expertise becomes imperative for thinking about how others “fit in” or are valued in collegiate athletics.

Black women ADIOs using their lived experience as an outsider in the “industry” (i.e., collegiate athletics) as a barometer for addressing the marginalization of others is evident in how Monique poses questions to herself:

Yeah, I think, being a part of two identities (race and gender) that have been historically underrepresented, marginalized, and oppressed in our society and in this industry as well, definitely is part of, like how I come into this space. I think it, it influences me and I'm always reflective of, um, “How would you feel if this happened to you?” Or, “How did you feel when it did happen to you?”

For Monique, Black women's “oppression” in collegiate sports [Borland and Bruening (12)] influences how she comes “into this space.” In the context of the dialogue, “space” is interpreted as ADIO leadership. Consequently, she centers this “oppression” and uses Black womanhood as a barometer for making sense of exclusivity, by asking, “How did you feel when it (i.e., marginalization) did happen to you?” Rather than detaching themselves from the lived experience of hostile intersectional invisibility (38), they lean into these experiences to center themselves on their perceptions of organizational inclusivity, in turn, creating a meaning-making of benign intersectional invisibility (38). Thus, they use their lived experience as an *outsider within* (29) to hold the *meaning* of expertise in their perceptions of organizational inclusivity.

### Intersectionality: Using lived raced-gendered experiences to make sense of organizational inclusivity

Although being a Black woman in the US social and sports systems can be an adverse experience due to issues of sexism and racism (6, 21), Black women ADIOs use these adverse intersectional realities to inform what it means to create inclusivity for others. Previous research discussed how Black women are hindered from opportunities in collegiate sports leadership due to their non-prototypical identities [Borland and Bruening (12)]. However, Black women ADIOs perceive their non-prototypical status (i.e., Black and woman) as informative for how they make sense of organizational inclusivity. Thus, their “intersectionalities (Serenity),” specifically the intersection of race and gender, are drawn upon as expertise to inform perceptions of organizational inclusivity. Jalyiah, on numerous occasions, discussed how her intersectional experiences empowered her to fulfill the job responsibilities of an ADIO and she perceived the intersectional realities of Black womanhood as giving her an upper hand in creating inclusivity for others. She shares:

And I just want to ensure that I can do everything I can to make sure that I am bettering the experience of those under my leadership, but then also for those who come after me. Um, I think from a professional perspective, we as Black women, have intersectional identities [entangled in many systems]. And think about some of those quotes about Black women “being the most disrespected person in America” [is relevant to my leadership].

The quote Jalyiah is alluding to are the words of Malcolm X, in which he states, “The most disrespected person in America is the Black woman. The most unprotected person in America is the Black woman. The most neglected person in America is the Black woman.” In the above quote, Malcolm X brought attention to the varying systems that disenfranchise Black women in the US and he acknowledged that Black women and Black men have similar, yet distinctive, marginalization. Jalyiah, drawing upon this quote after sharing how she aims to do “everything” she can to ensure she “betters the experience of [others],” expresses how, for her, creating inclusive organizations (i.e., her perception of organizational inclusivity) and using her experience as “the most disrespected person in America” to do so, are entangled in one another. Moreover, Jalyiah perceives her professional perspective on “bettering the experience of those under [her leadership]” as connected to Black women “having intersectional identities [entangled in many systems].” Like others in this study, Jalyiah's lived experience of marginalization holds the *meaning* of expertise, and such expertise is deployed as critical praxis (26) in the ADIO position and informs their perceptions of organizational inclusivity.

The participants provided specific examples of how their own adverse raced-gendered experiences (i.e., hostile intersectional invisibility) informed their perceptions of organizational inclusivity. Kayla shared how burdensome it is to comply or be pressured into complying with White patriarchal standards in the workplace as a Black woman (50). She discusses how her experiences of hostile intersectional invisibility (38) enable her “to be more cognizant of other people's identities.” Kayla asserts:

There are these two identities (race and gender) that I cannot change and people already may have an opinion about me because of these identities and realizing like that's a lot to carry, especially if you're like, I'm just trying to be good at whatever it is. And now I feel like I have to think about, “Okay, well I'm a woman in this space, so how am I dressing properly? I'm Black in this space? So is my hair appropriate? Like, can I say something like all of these things add up?” So, I feel like as I started to realize that the world isn't gonna always see *you*, even if you wanna be seen, I think that allowed me to be more cognizant of other people's identities.

For Kayla, the sexism (e.g., being concerned about how she is dressed) and racism [e.g., concerned about her hair being perceived as “appropriate” (read: professional)] is the simultaneity of marginalization that intersectionality brings attention to (25, 26). When sharing these experiences, from the onset she states, “There are these two identities that I cannot change,” which highlights that Kayla centers and draws upon the interconnected marginalization of both identities (i.e., race and gender), rather than one over the other (25). By doing so, she illustrates how both identities and their attached marginalization inform her perceptions of inclusivity, which for Kayla manifests as “be[ing] more cognizant of other people's identities.” Since the world does not see “[her]” and the intersection of the biases she navigates on the axis of race and gender (e.g., intersectionality), her perception of inclusivity takes a systemic perspective, as she continues to share how “intersectionality” enables the “understanding” (read: empathy, criticality, consciousness) necessary to conceptualize organizational inclusivity as considering how identities intersect to create marginalization entangled in more than one system (25, 26). She continues to display critical praxis (26), as she shares how her lived experiences as a Black woman enables her to question how the identity of others also intersects with multiple systems:

So I think those intersectionalities make it a big point because you're like, “Okay, if you can accept one part of me, how about the other part of me?” Um, so I think that those layers allow for you not to only think about gender and race, but then to think about, “Okay, what about sexuality? What about disability?" And really tie those into like, this is not just one part of someone like, yes, it is a piece of who they are, but like, you can't just ignore that part because it's, it's a larger part of who they are as a whole.

Kayla uses her understanding of Black women's relationship with patriarchy and racism to acknowledge and make sense of how individuals in her organization are also marginalized by more than one system (24, 25). Consequently, Kayla's perception of organizational inclusivity is to consider the “whole” person, that is, she considers the multiple systems that create exclusivity for them. Thus, she applies what she knows about intersectionality in a manner that demonstrates *critical praxis* (26). Keaton (16) discusses how the ADIO position is intended to create more diverse, inclusive, and equitable sports organizations. For Nia, such a responsibility “feels like what [her] lived experience is.” Nia asserts:

My identity as a Black woman informs my work because doing this work feels like what my lived experience is. Right? Like I have to help everybody else. I have to do the heavy lifting. I have to make myself smaller, right? To make this work bigger. Right? Um, so, it just, it, it kind of continues to reinforce who I am as a Black woman in this world and how I show up. Right? The two are hand in, the two are hand in glove.

Nia equates her responsibilities as an ADIO to “feel” like her lived experiences as a Black woman. But, upon making this comparison, she only shares the adverse realities of Black womanhood (e.g., hostile intersectional invisibility), like “mak[ing] herself smaller” (e.g., attempting to take up less space or be less visible) and “do[ing] the heavy lifting” [e.g., Strong Black woman stereotype/trope, see (51)]. ADIOs are espoused to be leaders who create more inclusive environments; hence, ADIOs *should* have a clear perspective on organizational inclusivity (16). Nia, drawing upon the adverse realities of Black womanhood (e.g., “doing the heavy lifting”) to “inform her work” (e.g., create more inclusive environments), is a picturesque example of Figure 1. Black women ADIOs draw upon their experience of racial and gendered marginalization to inform how they cultivate a shared perception of organizational inclusivity in their organization. This *means* that their own marginalization becomes perceived as an expertise in the ADIO position. Lastly, she concludes with an analogy, “…the two are hand in glove.” That is, the same way a glove fits a hand, the ADIO position fits Black women because the position and its associated responsibilities (e.g., cultivating a shared perception of organizational inclusivity) mean that Black women must be reflective of self and their marginalization, which makes their familiarity with marginalization become a tool for conceptualizing organizational inclusivity.

Previous scholarship studied how Black women administrators navigate racist and sexist organizational cultures because their identities are non-prototypical identities for collegiate sports leadership positions (12, 21, 27). Monique uses these lived experiences of “being stereotyped” in her leadership to ensure others can refrain from being on the “receiving end of [a] poor [organizational] culture.” She articulates:

Um, so it's personal, it's personal because, um, I've been on the receiving end of being stereotyped, being marginalized, being silenced. Um, on the, you know, receiving end of poor culture-... and negative culture. And that's why I do it, um, 'cause I don't want the people who come behind me in any of these positions, or any of these spaces to have to endure, or experience some of the things that I have.

Monique states “it's personal” two times, which seems to emphasize just how personally connected she feels to ensuring others can evade the marginalization she has experienced. Because she knows what a “poor” and “negative culture” looks and feels like, she centers these lived experiences to ensure they do not happen to others. Additionally, what she has “endure[d]” is the entangled nature of working in sexist and racist sports organizations (12). She shares, “And that's why I do it,” capturing how engaging in the work of ADIO leadership (read: cultivating a shared perception of organizational inclusivity in her organization) is rooted in her own intersectional marginalization as a Black woman in collegiate athletics (21) and society writ large (24, 35).

### Black feminist thought: Using black women's consciousness to make sense of organizational inclusivity

As participants discussed their ADIO leadership position, they alluded to Black women being uniquely equipped for the position, given that Black women have a “different lens,” are “inherently [different],” and “have a unique perspective.” I interpreted these proclamations as participants perceiving their intersectional identity as unlocking a particular consciousness, a Black feminist consciousness [or a Black Feminist Thought (BFT)] (24, 29) that informs their perceptions of organizational inclusivity.

Jalyiah speaks about this consciousness as if it is innate to Black women, as she asserts “…we (Black women) know what we're doing when we are advocating for that norm (read: exclusive practices/cultures) to be changed.” Nia brought attention to how the ADIO position is “hand in glove” with her lived experiences as a Black woman. Similarly, Jalyiah supports this notion by proclaiming that Black women in diversity leadership positions *just know* what they are doing because the job responsibility of creating inclusive organizations is a task that enables her to bring *herself* (lived experiences) into her job responsibilities of cultivating a shared perception of organizational inclusivity. Jalyiah states:

And therefore, we (Black women) know what we're doing when we are advocating for that norm (read: exclusive practice) to be changed. Um, we have a different lens of not fitting in, not having a voice, not being respected, having to fight up against again, all the systems that we're mentioning. We have just a very, very like unique intersectional lens with how we like wake up every morning and live our lives as women of color, Back women. Um, but then that directly translates into my ability to have a unique perspective and voice in our roles.

The consciousness that is developed from experiencing, navigating, and “fight[ing]” marginalizing systems is relevant to Jalyiah's ability to have a “unique perspective” in an ADIO position. Like other participants, she discusses how her lived experiences as a Black woman cannot be divorced from the realities of what an ADIO is *espoused* to do, which is to cultivate a shared organizational understanding of inclusivity (16). However, to do so, she centers her lived experiences to conceptualize what inclusivity is, something that Black women just “know” because of their familiarity with exclusivity (24, 25, 35). Kayla also operates from the standpoint of Black women *just knowing* how to create more inclusive organizations and she discusses how such a perspective is possibly “biased.” However, she questions if her biases are valid because her intersectional race-gendered experiences do give her a “unique way to connect with different people.” Kayla perceives connection as an attribute of organizational inclusivity, and she holds such a perspective because of her intersectional raced-gendered experiences that offer her a unique consciousness (read: BFT). Kayla shares:

I think that you have the – and this could be biased. But like, I think we (Black women) have, um, [a] unique way to connect with different people, um, because you, because of the intersectionality, right? Like, I feel like we can connect with the female student athletes, I also think we can connect with the [racially minorit[ized] population male or female.

Monique discusses how her experiences of being stereotyped as a Black woman give her the consciousness necessary (read: BFT) to be alert to how organizational spaces disregard those who have marginalizing experiences. Monique shares:

We (Black women), we... Many of us are stereotyped in the same way because we're talking about systems that operate in making assumptions about people. Systems that have made up their mind about Black women, regardless of where we come from. So, uh, many of those stereotypes follow us. So, when you think about the uniqueness of being in that room [read: in meetings with a majority White senior-level leadership], is that there are some shared and common experiences that, whether we've had them kind of personally, we know are happening in other spaces, and we're conscious of that.

Monique uses the consciousness that is derived from her own marginalizing experiences to acknowledge that her lived experiences are not happening in a vacuum. Her own oppression gives her the awareness and wherewithal to be conscious of how some organizational dynamics, create disadvantaged experiences for other marginalized communities as well. Consequently, her perception of organizational inclusivity considers how marginalizing experiences can occur due to the intersection of power, identity, and space. Nonetheless, this perspective is only developed from the consciousness that is unlocked from navigating “systems that have made up their mind about Black women.” Monique continues to build on this notion of her BFT, informing her perceptions of organizational inclusivity. She discusses:

And I think we (Black women) bring the perspective, um, because we inherently, like from a very basic level are different... than the people in the room. We have had to live in a world that we don't have the same privilege that a White male would have, and that has given us different experiences. And so, we're in a society that, um, views us differently.

Because Black women live in a world that does not afford them innate privileges based on their raced-gendered identities, they garnish “different experiences,” which bolsters their BFT (29, 35). Like Jalyiah, Monique also perceives Black women as *just knowing* what organizational inclusivity is because Black women are “inherently, like from a very basic level are different” and have vast experiences of exclusivity in organizations. Consequently, navigating collegiate athletics and their own respective athletic departments puts the BFT of Black women ADIOs into critical praxis (26), demonstrating how their adverse lived experiences are centered to conceptualize inclusivity, while also capturing how their lived experience and interpretation of these experiences come to hold the *meaning* of expertise for Black women ADIOs.

## Discussion

The Athletic Diversity and Inclusion Officer (ADIO) position is unlike any other position in collegiate athletic administration. It is the only position in which one's marginalization is drawn upon to meet the demands of the job responsibilities. ADIOs must hold a clear perception of organizational inclusivity (16), and Black women holding the position meet this demand by centering self and their own oppression of race-gendered marginalization they experience in collegiate athletics [Borland and Bruening (12)] and society writ large (35). Consequently, through the perceptions of Black women ADIOs, organizational inclusivity is defined as (a) creating contexts that do not mirror Black women's lived experiences, specifically as an *outsider within* mostly White athletic departments, (b) lived experiences entangled in systems of oppression, specifically sexism and racism, and (c) experiences that cultivate BFT in Black women, as this consciousness is bolstered through adverse realities of exclusion. The adverse experiences of Black women in sports being used as expertise to inform their perceptions of organizational inclusivity means that (a) these leaders consider the whole person and the varying systems that implicate their organizational experiences (read: intersectionality), (b) their leadership is attentive to who resides outside the margins of power (read: outsider within), (c) Black women ADIOs are conscious of how power and identity intersect to privy certain groups, and (d) they are attentive to how oppression can be shared but disparate with other marginalized groups.

These findings depict Black women ADIOs as leaders who engage with intersectionality at a level that demonstrates *critical praxis* (26). Collins and Bilge argue that problem-solving is a key aspect of using intersectionality as a tool of critical praxis. Black women ADIOs are attempting to solve the problem of organizational exclusivity in collegiate athletics (4, 9) by drawing upon their own marginalization. Consequently, Black women ADIOs in this study perceive their marginalization as a problem (or an issue) in sports organizations while also interpreting their marginalization as the solution. For example, by having a clear standpoint of their “own oppression” [(29) p. 747], these leaders draw upon their (unfortunate) expertise in experiencing exclusive and adverse organizational contexts to inform what they perceive organizational inclusivity to be. Therefore, the Black women ADIOs in this study are leading the charge of creating more inclusive sports organizations by centering on what inclusivity looks like for Black women.

Previous sports management scholarship has used intersectionality as a tool of critical inquiry and their participants are conscious of how their professional experiences are entangled in multiple systems of marginalization (12, 21). In this study, Black women ADIOs move beyond being aware of their social plight and enact this consciousness to inform their job responsibilities, which demonstrates critical praxis (26). These inaugural organizational leaders use their hostile intersectional invisible experiences (38) as expertise (see Figure 1). Therefore, the adverse lived experiences of Black women ADIOs operate as both hostile intersectional invisibility and benign intersectional invisibility (38). Unbeknown to participants, while sharing their hostile intersectional invisibility experiences, they also offer insight into their perceptions of organizational inclusivity. Hence, within the same syllogism, Black women ADIOs can flip their adverse experiences into being systemically and structurally focused diversity leaders: a picturesque display of benign intersectional invisibility (38).

Although Black women ADIOs can transform their marginality into valued and recognized expertise, future scholarship must explore how this expertise contributes to transforming their sports organization. Because this contradictory location as experts is drawing upon critical knowledge from the margins, future research should study how this knowledge is granted decision-making power to create substantive organizational change. Otherwise, their expertise is conditional.

This scholarship adds to and complicates the literature on Black women collegiate sports administrators. In one sense, Black women ADIOs are marginalized by the same systems of power that hinder the career ascension of Black women coaches and ADs (12, 21, 27). However, Black women coaches and ADs have disparate formalized job responsibilities in comparison to ADIOs (4, 16). Currently, the utility or praxis of intersectionality has not been documented in the same manner for coaches and ADs as it has for diversity leaders in this study. Serenity, Nia, Kayla, Jalyiah, and Monique perceived their understanding of intersectionality to elevate their ability to create inclusive organizations, which is their primary and formal job responsibility (16). Black women coaches and ADs have yet to be documented using their “own oppression” to complete their formal job responsibilities.

Given that Black women in sports are conscious of their marginalization, moving forward, scholars must be attuned to how they make sense of this oppression and if they possibly *use* their marginalization, like the Black women ADIOs in this study. We, as a scholarly community, have done a commendable job highlighting how intersectionality is a valuable tool of critical inquiry that foregrounds the entangled systems Black women in sports navigate. Future work should not merely report these barriers that we know to exist but also examine what these barriers *mean*, how these barriers correlate to their mental health, or why Black women in sports remain in the profession despite these systems of marginalization. Moving forward, sports phenomena-specific applications of intersectionality can more explicitly discuss how the theoretical framework is a powerful tool for understanding (critical inquiry) and addressing (critical praxis) issues of organizational inclusivity (26).

Lastly, although not explicitly shared, one can infer from the excerpts how navigating such hostility must have an adverse effect on participants' mental health and ability to remain committed to the work of creating diverse, equitable, and inclusive athletic departments. As the ADIO position continues to become more widely adopted, we need not only investigations of how Black women *do* this work but also examinations of how they take care of themselves while engaging in this work.

## Data availability statement

The datasets presented in this article are not readily available because Data will not be uploaded, shared, or accessible to the public. Requests to access the datasets should be directed to ajhanai.keaton@louisville.edu.

## Ethics statement

The studies involving human participants were reviewed and approved by University of Connecticut Institutional Review Board. The patients/participants provided their written informed consent to participate in this study.

## Author contributions

The author confirms being the sole contributor of this work and has approved it for publication.

## Conflict of interest

The author declares that the research was conducted in the absence of any commercial or financial relationships that could be construed as a potential conflict of interest.

## Publisher's note

All claims expressed in this article are solely those of the authors and do not necessarily represent those of their affiliated organizations, or those of the publisher, the editors and the reviewers. Any product that may be evaluated in this article, or claim that may be made by its manufacturer, is not guaranteed or endorsed by the publisher.
